# Higher Levels of Cardiovascular Biomarkers Following Hypertensive Compared to Normotensive Pregnancy

**DOI:** 10.1002/pmf2.70064

**Published:** 2025-09-12

**Authors:** Austin M. Gabel, Lindsay Cheu, Mindy Pike, Kelsey L. Olerich, Alisa Kachikis, Stephen A. McCartney, Raj Shree

**Affiliations:** ^1^ Medical Scientist Training Program University of Washington Seattle Washington USA; ^2^ Department of Obstetrics and Gynecology University of Washington Seattle Washington USA

**Keywords:** biomarkers, cardiovascular disease, cardiovascular risk, hypertensive disorders of pregnancy

## Abstract

Hypertensive disorder of pregnancy (HDP) is associated with an increased risk for later‐life cardiovascular disease (CVD). Whether the HDP pregnancy itself confers risk toward CVD later in life is suggested in several epidemiologic studies. Given this connection and that the HDP exposure itself may play a role, understanding whether markers associated with cardiovascular risk vary based on HDP history in the years following pregnancy may assist with risk stratification and development of targeted interventions. We measured 77 proteins (CVD‐associated and inflammatory markers) in *n* = 22 individuals with a history of HDP and *n* = 42 matched controls with no HDP history at a median of 4 years after pregnancy. Several CVD‐associated proteins (fibrinogen, fetuin‐A, L‐selectin, and alpha‐1‐acid glycoprotein) were significantly elevated, by orders of magnitude, in individuals with a history of HDP compared to normotensive pregnancies (all *p* < 0.0001). In multivariable linear regression models controlling for age, body mass index, chronic hypertension, and diabetes, a history of HDP remained associated with higher levels of CVD‐associated proteins (all *p* < 0.0001). We clustered samples based on global patterns of CVD protein expression and found a significant difference in CVD protein expression patterns between post‐Normal and post‐HDP samples. Conversely, differences in circulating inflammatory markers were largely insignificant or more subtle than those observed with the CVD‐associated proteins. Identification of biomarkers associated with CVD in the intervening years after HDP but before evident CVD is critical to understanding post‐HDP cardiovascular risk to provide insight for the development of therapeutic interventions that mitigate CVD event risk in this high‐risk population.

## 1 INTRODUCTION

Cardiovascular disease (CVD) is the leading global cause of death and sex‐based differences exist with respect to presentation and risk factors [1, 2, 3]. In women, reproductive factors may partially explain these differences [2, 4]. Several epidemiological studies demonstrate a strong link between hypertensive disorder of pregnancy (HDP), such as preeclampsia, and an increased risk for later‐life CVD, including coronary artery disease, myocardial infarction, heart failure, and stroke [5, 6, 7, 8, 9, 10]. This risk is further multiplied by recurrent episodes of HDP and preterm HDP [5]. The mechanisms contributing to increased CVD risk following HDP are poorly understood. Although underlying risk factors (e.g., obesity, diabetes, chronic hypertension) likely play a strong role [11], epidemiologic and some translational data suggest that the HDP pregnancy itself may be an independent risk factor for CVD development [6, 8, 9, 12]. Regardless of the mechanism, methods of identifying individuals at high‐risk for CVD after an HDP pregnancy are lacking. Although national societies include history of HDP in their algorithm for CVD risk classification [13], formal evidence‐based guidelines for long‐term screening, monitoring, or interventions do not exist due to a poor understanding of the clinical and molecular trajectory following HDP but before evident CVD [14].

Although the clinical symptoms of HDP overwhelmingly resolve following delivery, studies demonstrate that the characteristic vascular dysfunction of HDP persists, over the short (1–3 months) and long‐term period (up to 15 years after delivery) [15, 16, 17, 18]. Individuals with a history of HDP have attenuated cerebral vascular reactivity [19], increased arterial stiffness [20, 21, 22], and increased carotid‐intima thickness [23, 24, 25], years after delivery. Additionally, subclinical cardiac impairment can be detected after HDP [26, 27]. A key study of a cohort just 2–7 years (median 3 years) after their first pregnancy noted a higher rate of hypertension in those with recent pregnancies complicated by HDP, with over a fourfold greater risk when HDP was accompanied by indicated preterm birth [28]. Another study found that elevated third‐trimester blood pressure was associated with dyslipidemia 10–14 years after pregnancy [29]. Although clinical CVD largely occurs many years after HDP, subclinical changes are likely evident prior to this. Thus, the intervening years present a unique time for risk‐stratification and consideration of therapeutics.

Mechanistic studies have recently begun to understand the pathways contributing to persistent vascular changes following HDP that may ultimately lead to CVD [30, 31]. As inflammation is an overlapping phenomenon between HDP and CVD [32, 33, 34, 35, 36, 37], the role of inflammatory markers in explaining this connection is of interest [38]. Additional pathways that may contribute to altered vascular function, however, remain understudied. Critical to our understanding of long‐term CVD risk following HDP are investigations into molecular signatures in the years after the HDP pregnancy. We sought to assess levels of circulating plasma proteins associated with inflammation and cardiovascular risk among those with a prior history of HDP compared to those with no HDP history. We hypothesize that CVD‐associated and inflammatory proteins will be elevated in those with HDP history compared to those with no HDP history.

## 2 METHODS

### 2.1 Study participants

We prospectively recruited a convenience sample of non‐pregnant participants who had sought delivery care at our hospital. Cases included those with history of HDP in any pregnancy, while controls had no pregnancy history of HDP. HDP was confirmed by medical record review (by KO and SS) using current clinical criteria [39]. All participants underwent written informed consent through an approved Institutional Review Board protocol (STUDY00001636). Among these non‐pregnant participants, we identified *n* = 22 as having a history of HDP in a prior pregnancy (cases). Controls (*n* = 42) were identified by matching cases (approximately 1:2) to interval since most recent pregnancy. We did not match on any other demographic factors such as age, body mass index (BMI), chronic hypertension, or diabetes. Participants were not pregnant and presented for outpatient peripheral blood draws and were free of any acute illness at the time of sampling. Given the nature of the study, some demographic variables were assessed at the time of sample collection (age, parity, time since most recent pregnancy [all participants], time since most recent HDP pregnancy [cases only]), while others were ascertained from medical record review of the most recent pregnancy (all participants) or the most recent HDP pregnancy (cases only), and included BMI, gestational age at delivery, neonatal birthweight, Cesarean delivery, and neonatal sex. Some variables are static, including self‐reported race and ethnicity. We did not exclude participants if they had any history of chronic hypertension or diabetes (pre‐existing or history of gestational diabetes) from either group.

### 2.2 Sample collection and processing

Peripheral blood was collected in acid citrate dextrose solution A‐vacutainer tubes and underwent processing (unfrozen) within 24 h (majority within 12 h). Plasma was isolated by centrifugation of whole blood for 10 min at 1500 g and stored at −80°C. All samples were run through the multiplex assay in the same batch.

### 2.3 Multiplex assay

We collected single‐time‐point plasma samples from each participant and submitted aliquots for multiplex LASER assay protein quantification [40]. This multiplex technology relies on color‐coded polystyrene beads, minimizing sample volume needs while maximizing the number of measures per sample. The bead analyzer (Bio‐Plex 200) includes a dual‐laser system and a flow‐cytometry system. One laser activates the fluorescent dye within the beads, which identifies the specific analyte. The second laser excites the fluorescent conjugate (streptavidin‐phycoerythrin) that has been bound to the beads during the assay. The amount of the conjugate detected by the analyzer is in direct proportion to the amount of the target analyte. The results are quantified according to a standard curve, which provides a large range at which protein levels can be quantified (e.g., the standard curve for BNP is 22.86 pg/mL to 50,000 pg/mL). Individual protein measures with values out of range based on assay parameters were excluded.

In all participants, we measured a total of 77 proteins encompassing three distinct custom protein panels—CVD‐associated proteins (*n* = 15), immune‐related cytokines (*n* = 47), and soluble cytokine receptors (*n* = 15) (Table 1). These custom protein panels were chosen based on an understanding of the cardiovascular underpinnings of HDP [33, 41, 42], its implications on later‐life CVD risk [7, 18, 20, 22, 27], and that others have reported immune factor alterations after HDP pregnancy [38, 43, 44].

**TABLE 1:** Circulating proteins measured in plasma via multiplex assay.

Protein panel	Individual proteins measured
CVD‐associated proteins	BNP, NTproBNP, CK‐MB, CXCL6/LIX/GCP‐2, CXCL16, Cardiac Troponin I (cTnI), α2‐Macroglobulin, AGP, CRP, Fetuin A, Fibrinogen, Haptoglobin, L‐Selectin, PF4, SAP
Cytokines and chemokines	sCD40L, EGF, Eotaxin, FGF‐2, Flt‐3 ligand, Fractalkine, G‐CSF, GM‐CSF, GROα, IFNα2, IFNγ, IL‐1α, IL‐1β, IL‐1ra, IL‐2, IL‐3, IL‐4, IL‐5, IL‐6, IL‐7, IL‐8, IL‐9, IL‐10, IL‐12p40, IL‐12p70, IL‐13, IL‐15, IL‐17A, IL‐17E/IL‐25, IL‐17F, IL‐18, IL‐22, IL‐27, IP‐10, MCP‐1, MCP‐3, M‐CSF, MDC (CCL22), MIG, MIP‐1α, MIP‐1β, PDGF‐AA, PDGF‐AB/BB, RANTES, TGFα, TNFα, TNFβ, VEGF‐A
Soluble cytokine receptor	sCD30, sEGFR, sgp130, sIL‐1RI, sIL‐1RII, sIL‐2RA, sIL‐4R, sIL‐6R, sRAGE, sTNFRI, sTNFRII, sVEGFR1, sVEGFR2, sVEGFR3

### 2.4 Statistical analysis

Demographic characteristics between the post‐HDP and post‐Normal pregnancy participants were compared using χ^2^ tests, Student's *t*‐tests, and Wilcoxon rank‐sum tests. Differences in CVD‐associated proteins, immune‐related cytokines, and soluble cytokine receptors between the two groups were examined using Wilcoxon rank‐sum tests. The log_2_(fold‐change) between post‐HDP and post‐Normal pregnancy samples were plotted against significance from Wilcoxon rank‐sum tests in a Volcano plot. A Bonferroni corrected *p* value of < 0.0006 was considered significant for individual tests between post‐HDP/post‐Normal and each protein. After identifying the top four most significantly elevated proteins, we performed linear regression to examine the association between HDP status (post‐HDP vs. post‐Normal) and protein levels. We first completed univariable linear regression and then used multivariable linear regression adjusted for maternal age at sample collection, BMI, diabetes, and chronic hypertension. For each variable, we determined the univariate (unadjusted) or multivariate coefficient, reported as the log_10_(coefficient). We also performed sensitivity analyses by removing individuals with chronic hypertension or diabetes, given that these conditions may impact biomarker measures.

Global patterns among CVD‐associated proteins were investigated using *Z*‐scores and principal components analysis. Summary *Z*‐scores were calculated by summing *Z*‐scores for all 15 CVD‐associated proteins among all individuals and compared between post‐HDP and post‐Normal pregnancy participants using a two‐sided Wilcoxon rank‐sum test. We assessed similarity of samples by constructing a heatmap and clustering samples using default settings in *pheatmap*, which utilizes an agglomerative hierarchical clustering algorithm [45]. In short, the global expression profile of measured proteins was compared to determine how similar the samples were. We then simulated data to build an empiric distribution of the number of post‐HDP samples we would expect from random data to compare to the distribution in our observed samples. To quantify intragroup heterogeneity, we completed Pearson correlation by assessing expression of all proteins of interest in one sample relative to each other sample within the group of interest (e.g., compare protein expression of all CVD proteins in patient 1 to that in patient 2, and so forth). All statistical analyses were completed in R Studio Version 2023.09.1+494 utilizing base functions along with the packages *tidyr* [46], *PCAtools* [47], *ggplot2* [48], *ggfortify* [49], *broom* [50], and *wesanderson* [51]. A *p* value less than 0.05 was considered statistically significant for linear regression analyses and summary measures compared across post‐Normal and post‐HDP groups. Similar analyses were used to examine the patterns and clustering for immune‐related cytokines.

In a secondary analysis, we examined the association between protein levels of fibrinogen and L‐selectin and the time interval since last pregnancy. The mean plasma protein concentration per group (post‐HDP or post‐Normal) was taken at each time point and the correlation with the time interval since last pregnancy was examined using Pearson correlation coefficients.

## 3 RESULTS

### 3.1 Study cohort demographics

**FIGURE 1:**
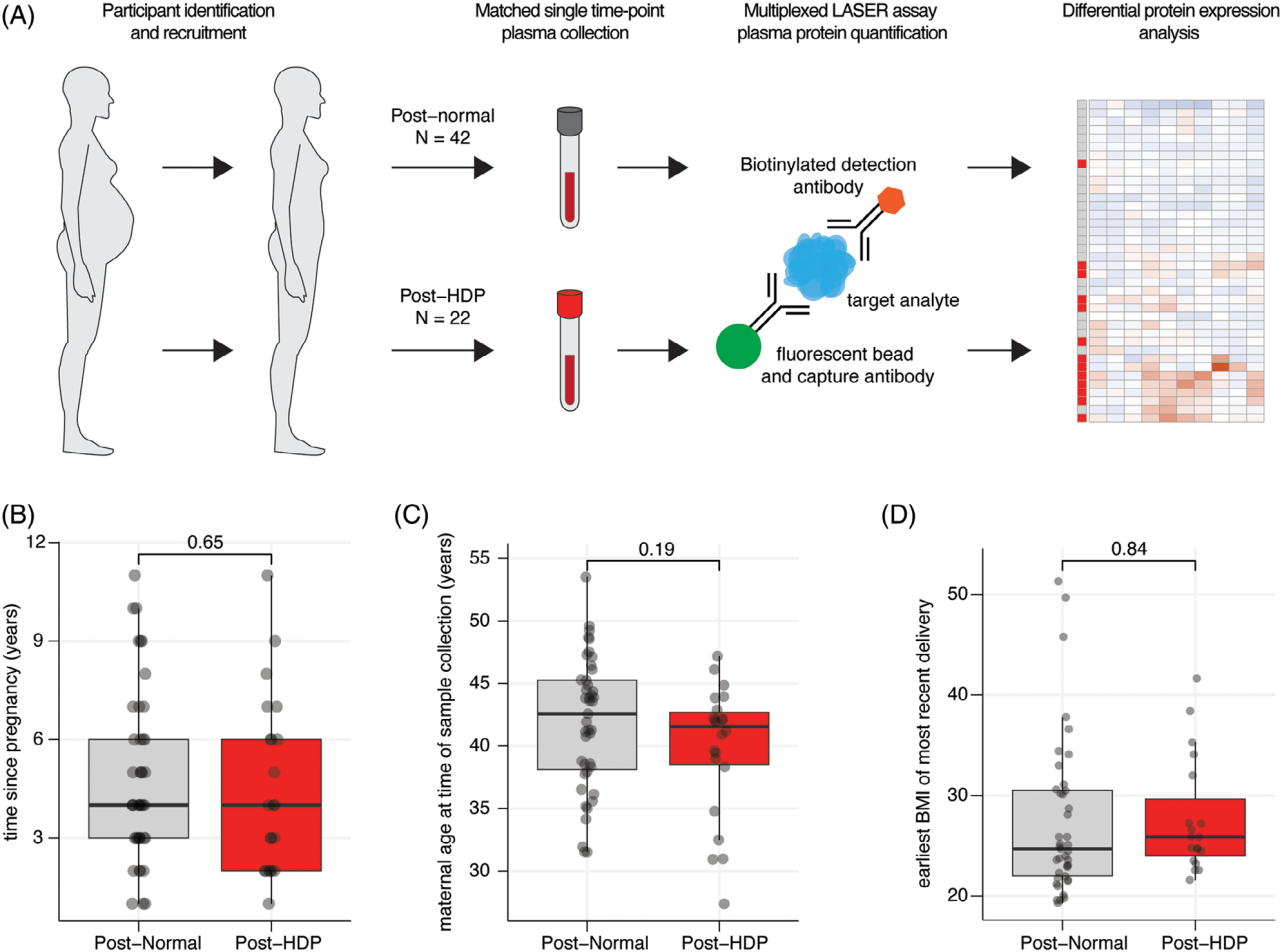
Workflow and relevant clinical parameters of the study cohort. (A) Schematic of sample collection, multiplex assay, and analysis. Samples were collected at a single time point and matched for time since last pregnancy. Plasma was submitted for multiplexed LASER assay protein quantification included in pre‐assembled panels. (B–D) Boxplots comparing time since last pregnancy (B), maternal age at the time of sample collection (C), and earliest body mass index (BMI) of most recent delivery (D) between post‐Normal and post‐HDP groups. For all, the *p* value is from a two‐sided Wilcoxon‐Rank Sum test.

The overall study design is outlined in Figure 1A. We included a total of 64 participants (*n* = 22 post‐HDP and *n* = 42 post‐Normal pregnancy). Among the post‐HDP group, 5 (22.7%) had a history of gestational hypertension, 3 (13.6%) had a history of preeclampsia without severe features, 12 (54.5%) had a history of preeclampsia with severe features (inclusive of superimposed preeclampsia and HELLP syndrome), and 2 (9.1%) had postpartum preeclampsia. Although we did not match on age, BMI, chronic hypertension, or diabetes, there were no significant differences between groups with respect to these parameters and we adjusted for these clinical parameters in regression analyses. No difference in time since pregnancy confirms appropriate matching (Table 2, Figure 1B–D). As expected, those with HDP delivered at earlier gestational ages and infants of lower birthweight (Table 2).

**TABLE 2:** Demographics of the study population.

	Post‐HDP (*n* = 22)	Post‐Normal (*n* = 42)	*p* value
Age at sample collection (years) *Missing: n = 0*	39.7 ± 5.3	41.7 ± 5.3	0.16
White race *Missing: n = 0*	19 (86.4)	34 (80.9)	0.59
Hispanic ethnicity *Missing: n = 1*	4 (18.2%)	2 (4.8)	0.08
Parity *Missing: n = 0*	2 (1–2)	2 (1–2)	0.36
Time since most recent pregnancy (years) *Missing: n = 0*	4 (2–6)	4 (3–6)	0.54
Time since HDP pregnancy (years) *Missing: n = 0*	6 (4–8)	N/A	N/A
Chronic hypertension^b^ *Missing: n = 0*	3 (13.6)	5 (11.9)	0.84
Diabetes^a^ *Missing: n = 0*	5 (22.7)	7 (16.7)	0.55
Body mass index (kg/m^2^)^c^ *Missing: n = 5*	25.9 (23.5–32.0)	24.8 (21.0–30.5)	0.34
Gestational age of delivery (weeks)^c^ *Missing: n = 2*	37.6 (32.4–39.0)	39.6 (38.6–40.2)	< 0.001
Neonatal birthweight (grams)^c^ *Missing: n = 1*	2572 (1877–3198)	3297 (3113–3651)	< 0.01
Cesarean delivery^c^ *Missing: n = 0*	12 (54.5)	17 (40.5)	0.28
Female neonatal sex^c^ *Missing: n = 0*	10 (45.4)	17 (49.5)	0.78

*Note*: Data are *N* (%), mean (± standard deviation), median (interquartile range).

Abbreviation: HDP, hypertensive disorders of pregnancy.

^a^ History of gestational diabetes (in any pregnancy), type 1 diabetes, or type 2 diabetes.

^b^ At the time of pregnancy or at the time of sample collection.

^c^ Of HDP pregnancy for the Post‐HDP group and of the most recent pregnancy beyond 20 weeks gestation for the Post‐Normal group.

### 3.2 CVD‐associated proteins are elevated following an HDP pregnancy

**FIGURE 2:**
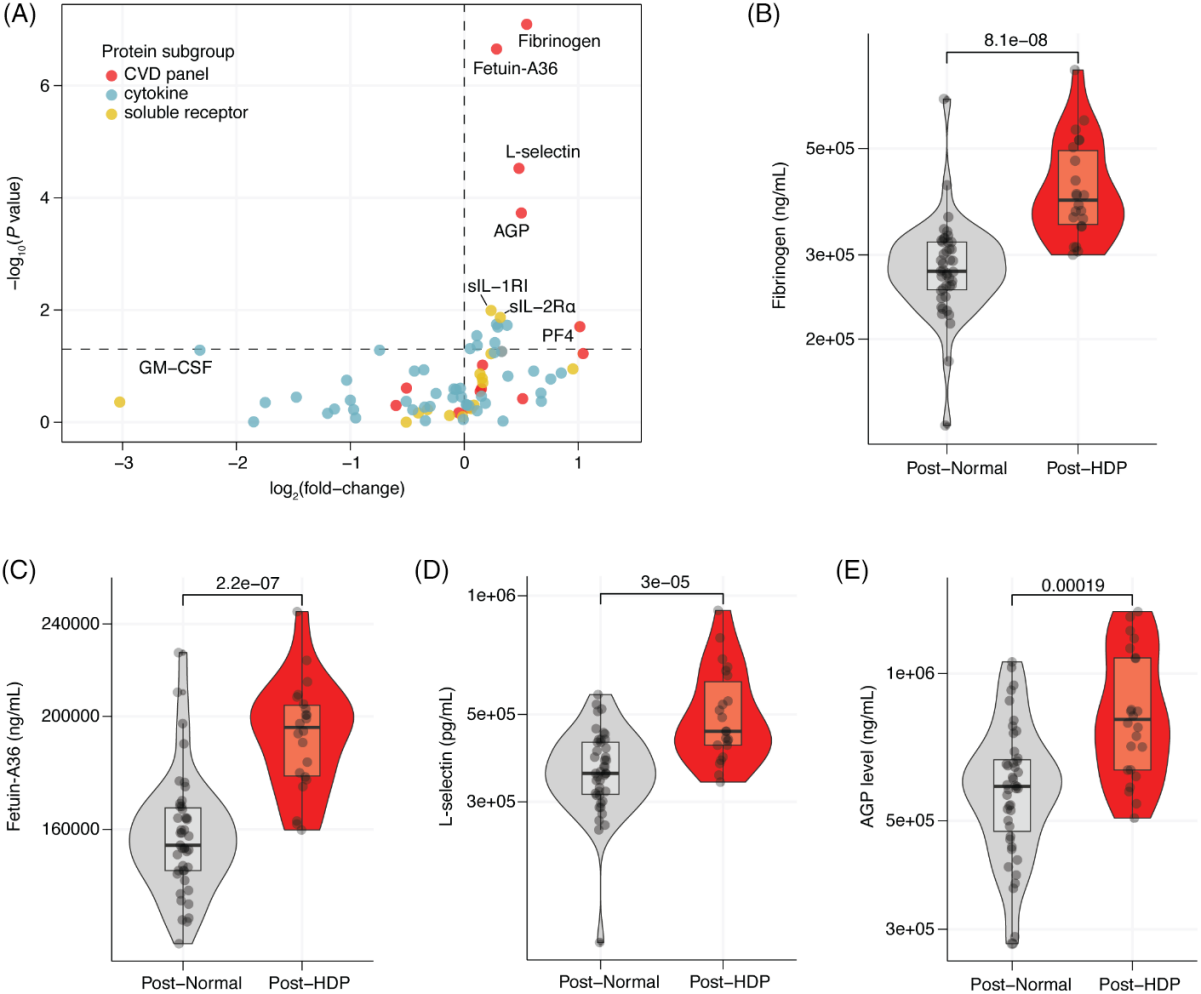
Several CVD‐associated proteins are elevated in plasma in the few years after an HDP pregnancy. (A) Volcano plot of all 77 assays plasma proteins colored by protein subset (red, CVD panel; blue, cytokine; and yellow, soluble receptor) with the log_2_(fold‐change) in protein levels between post‐HDP and post‐Normal and the −log_10_(*P* value) from a two‐sided Wilcoxon rank sum test. (B–E) Violin plots of fibrinogen (B), fetuin‐A36 (C), L‐selectin (D), and AGP (E) protein levels in plasma from individuals following a normotensive pregnancy (Post‐Normal) or pregnancy complicated by HDP (Post‐HDP). For all, the *p* value is from a two‐sided Wilcoxon rank sum test.

Among all 77 measured proteins, fourteen proteins had significantly higher expression in the post‐HDP group (Figure 2A). Among these enriched proteins, the top four proteins—fibrinogen, fetuin‐A36, AGP (alpha‐1‐acid glycoprotein), and L‐selectin—are all CVD‐associated proteins (Figure 2B–E). None of the measured proteins were significantly downregulated in the post‐HDP cohort compared to controls.

For the top enriched plasma proteins (fibrinogen, fetuin‐A36, L‐selectin, and AGP), a history of HDP was significantly associated with plasma protein levels after adjustment for maternal age at sample collection, earliest BMI of the most recent pregnancy, chronic hypertension, and diabetes (Figure S1A–D). The estimated coefficient for post‐HDP was similar between the univariable and multivariable analysis for each protein, suggesting that among the covariates included in our analysis, HDP history largely explains the elevated plasma protein levels observed in our cohort (Figure S1E–H; Table S1). Exclusion of individuals with chronic hypertension or diabetes in sensitivity analyses did not change our results (all *p* < 0.001, results not shown).

### 3.3 Globally, CVD‐associated proteins are significantly elevated in the post‐HDP samples

**FIGURE 3:**
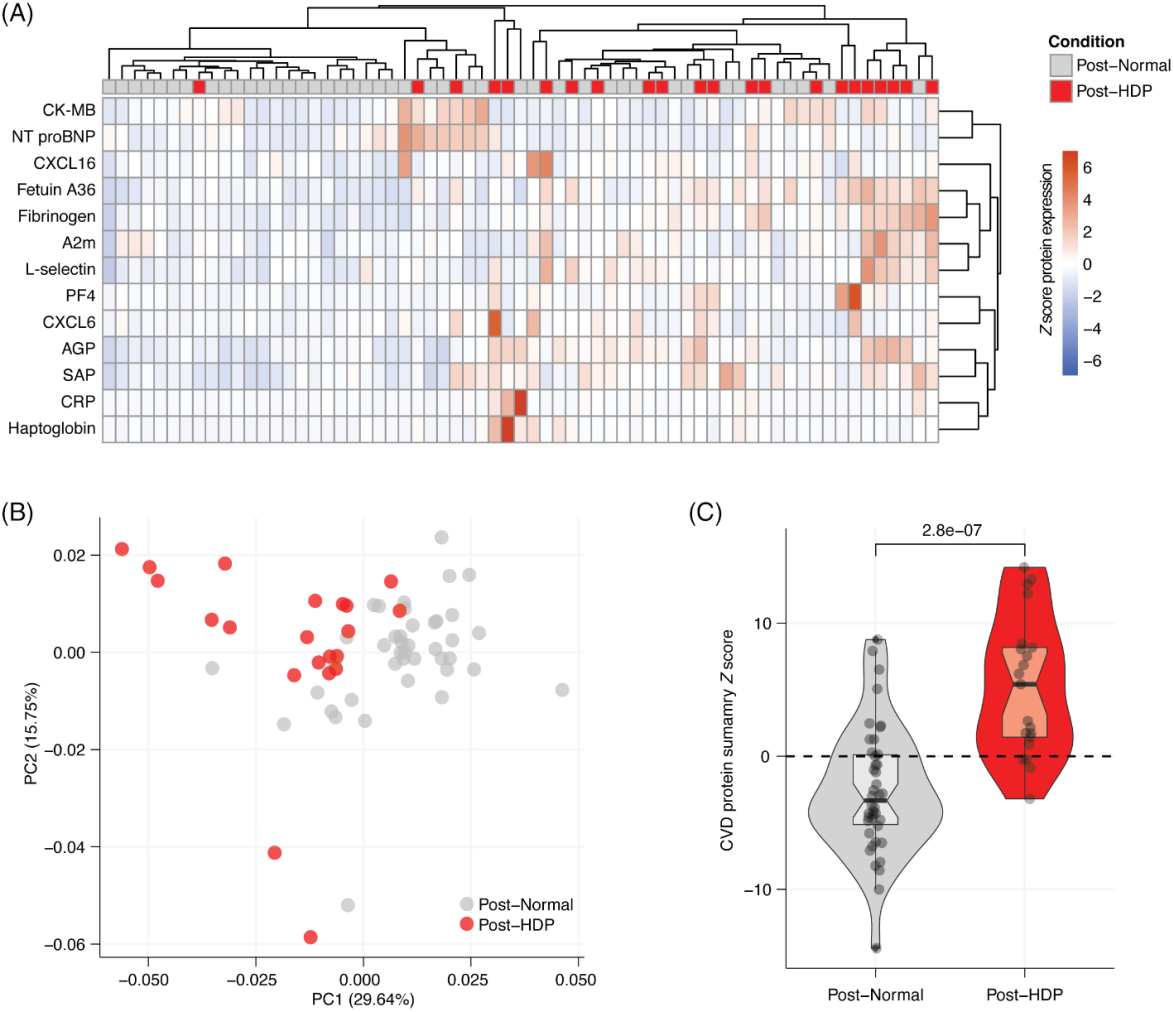
Global patterns of CVD‐associated proteins in the few years after an HDP or normotensive pregnancy. (A) Heatmap of *Z* scores of CVD panel protein expression with rows reflecting an individual protein and columns corresponding to an individual participant sample. Samples colored by condition as Post‐Normal (gray) or post‐HDP (red). (B) PCA plot of Post‐Normal (gray) and Post‐HDP (red) protein values for plasma protein expression values of 15 CVD‐associated proteins. (C) Violin plot of CVD summary *Z* score calculated by summing *Z* scores of all 15 CVD‐associated protein levels in plasma from individuals following a normotensive pregnancy (Post‐Normal) or pregnancy complicated by HDP (Post‐HDP). *P* value from a two‐sided Wilcoxon rank sum test.

In addition to the individual proteins listed above, we investigated global patterns among all the CVD‐associated proteins (*n* = 15) in our cohort. Heatmap of *Z*‐scores (Figure 3A) and principal component analysis (PCA) (Figure 3B) of the CVD‐associated proteins demonstrates reasonable, but not perfect, separation of the population based on HDP history. A summary *Z*‐score demonstrated that CVD‐associated proteins are globally elevated in the post‐HDP group compared to the post‐Normal participants (*p* < 0.0001) (Figure 3C). This indicates that exposure to HDP has variable effects on plasma protein levels; yet individuals that experienced an HDP display significant elevations in CVD‐associated plasma proteins in aggregate in the few years after an HDP pregnancy.

### 3.4 Significant heterogeneity exists among post‐HDP samples

When comparing global patterns in protein expression, we observed substantial heterogeneity in CVD‐associated protein levels among individual samples (Figure 3A,B). Simulated data that clustered the samples first split the 64 samples into two groups of 32 samples, where cluster 1 contained 15.6% (5/32) and cluster 2 contained 53.1% (17/32) post‐HDP samples, respectively. We then built an empiric distribution of what fraction of post‐HDP samples we would expect to see if 32 samples were selected at random (Figure S2A,B). The observed results were more extreme than 99.9% and 99.7% of all simulated data, for cluster 1 and cluster 2, respectively, strongly suggesting the clustering we observe is not due to random chance (Figure S2B).

We then quantified levels of heterogeneity among measured samples. We completed pairwise Pearson correlations of the plasma concentrations of CVD‐associated proteins, comparing each individual plasma sample to every other plasma sample in our study (Figure S2C). We found that post‐Normal samples were significantly more similar to each other, reflected in a more positive average Pearson correlation coefficient (*R*), than post‐HDP samples were to each other (Figure S2D).

### 3.5 CVD‐associated proteins in the period after pregnancy

As part of a secondary analysis, we evaluated the relationship between time since most recent pregnancy and plasma protein levels by quantifying the mean plasma protein concentration per group (post‐Normal or post‐HDP) at each year. We observed a significant negative correlation between fibrinogen levels and interval since last pregnancy among post‐HDP participants, but not in those with a history of a normotensive pregnancy (Figure S3A). In comparison, plasma L‐selectin levels negatively correlated with time since last pregnancy in both groups; however, levels were consistently higher in individuals with a history of HDP compared to those with a history of normotensive pregnancies (Figure S3B). Other CVD‐proteins did not demonstrate any notable differences across time (data not shown).

### 3.6 Inflammatory markers did not vary in the few years after HDP

We did not detect circulating inflammatory or soluble cytokine receptors to be as substantially altered as CVD‐associated proteins in the few years following HDP (Figure 2A). Several inflammatory proteins were higher in the post‐HDP population (sIL‐1RI, sIL‐2Rα, M‐CSF, RANTES, MDC, and IL‐12p40), but they did not reach significance after adjusting for multiple comparisons (Figure S4A–F). When assessing global patterns of inflammatory protein expression in plasma samples, we did not detect obvious clustering of post‐HDP samples via dendogram or using principal component analysis (Figure S4G‐I). Though no inflammatory proteins were significantly elevated (after adjustment for multiple comparisons), a summary *Z* score measuring the aggregate expression of all inflammatory proteins was significantly elevated in the post‐HDP samples compared to post‐Normal samples (Figure S4I). This suggests that while few of the measured inflammatory proteins are significantly elevated in the few years following HDP, overall levels of inflammation may be significantly elevated in the few years following HDP.

## 4 DISCUSSION

Here we identify several plasma proteins elevated in the few years following an HDP pregnancy. The most significantly enriched are those relevant to CVD (fibrinogen, fetuin‐A, L‐selectin, and AGP). In several vascular disease pathologies, high fibrinogen predicts poor outcomes, including in peripheral arterial disease, myocardial infarction, and ischemic stroke [52]. Higher levels of fetuin‐A was associated with increased risk of ischemic stroke and myocardial infarction in a relatively young population (< 57 years old) [53]. Interestingly, at the same plasma level of fetuin‐A, the risk of these adverse cardiovascular outcomes was higher among women than men [53]. Although elevated L‐selectin levels are associated with smoking [54], prior studies have noted mixed results regarding an association with CVD [55, 56]. It is, however, proposed that circulating L‐selectin may serve an anti‐inflammatory function with respect to atherosclerotic plaque formation [55]. AGP, also known as orosomucoid, is associated with the presence of carotid plaques [57], ischemic stroke [57, 58], and arterial stiffness [59]. We have identified some of these same circulating markers to be elevated in the few years (median 4 years) after an HDP pregnancy.

Emerging data indicate that the HDP pregnancy itself may confer risk toward later‐life CVD [6, 8, 9, 12]. Additionally, individuals with HDP history develop CVD along an accelerated pathway with events occurring more frequently in their 5th and 6th decades compared to their counterparts [6]. Our data suggest that after accounting for several critical risk factors associated with CVD (BMI, diabetes, chronic hypertension) that those with a history of HDP demonstrate an adverse pre‐clinical cardiovascular profile that may be a harbinger of increased later‐life CVD risk. Few studies have measured post‐pregnancy CVD‐associated proteins, both in the short term (e.g., BNP six weeks postpartum [60]) and long term (high‐sensitivity cardiac troponin I at 9–10 years post‐pregnancy [61]), and have found no significant differences among those with and without HDP history. Our study aligns with these prior findings with respect to these specific proteins, however, also suggests that a broader range of CVD‐associated biomarkers warrant further investigation in larger cohorts.

We did not observe differences in individual inflammatory markers; however, persistent inflammation following an HDP pregnancy is reported in some studies [38]. It is possible that we were underpowered to detect differences and that these results require further contextualization with cellular immune data. When assessing inflammatory markers in aggregate, we did detect a significant elevation in post‐HDP samples compared to controls. Collectively, this could indicate persistent low‐level inflammation following HDP, or elevations in inflammatory markers not included in our study. Our results, however, suggest that CVD‐associated proteins may be more robust biomarkers than inflammatory mediators for inclusion in future prospective studies.

Few studies have investigated circulating biomarkers in the years after the HDP pregnancy. In those with HDP, one study demonstrated increased TNF‐ɑ three months postpartum [62] and another noted an increased IL‐6/IL‐10 ratio 20 years after the index pregnancy [63]. A recent study that evaluated some cardiovascular (VCAM‐1, VEGF, CD40L, GDF‐15, and ST‐2) and metabolic biomarkers in individuals 5–10 years after index pregnancy did not find an association between HDP and these biomarkers [64]. We also noted no difference in some of these same markers (VCAM‐1, VEGF, CD40L), however, uncovered well‐established CVD‐associated proteins (fibrinogen, fetuin‐A, L‐selectin, AGP) to be elevated in the few years following HDP that were not measured in the above‐listed study. We also observed heterogeneity in the levels of these CVD‐associated proteins in the post‐HDP cohort. This may support a more recent understanding of multiple phenotypes of preeclampsia giving insight into the possibility of developing a unique CVD outcome based on the HDP phenotype [65].

Our study is not without limitations. We only had access to samples at a single time point; thus, our analysis of the changes in plasma protein levels by interval from pregnancy (Figure S3A,B) requires validation with serial sampling from the same individual. For example, fibrinogen is an acute‐phase reactant [66, 67, 68, 69], and as our participants presented as outpatients, non‐pregnant, and without evidence of any acute illness, further work is needed to clarify the etiology of this elevation in those with a history of HDP in the absence of known causes. We also lack pre‐pregnancy measures to definitively determine whether the HDP exposure plays a role in leading to increased levels of these CVD‐associated proteins or whether these elevations were present before the pregnancy. Additionally, we do not have blood pressures or antihypertensive medication use at the time of the blood draw, and this may be responsible for some of the observed differences. Conversely, pre‐pregnancy measures are challenging to obtain as recruitment of thousands of individuals is necessary to capture enough HDP cases, particularly those with severe phenotypes carrying the highest risk for later‐life CVD [5]. Our sample size precluded stratified analyses based on HDP severity. Lastly, our population lacks adequate representation from American Indian and Alaskan Native and African American pregnant individuals, acknowledging that they experience the highest rates of HDP in the United States (US) [70].

Strengths of our study include the use of a highly sensitive multiplex assay that can measure numerous proteins with minimal sample volume. Employment of this expansive approach in this rare cohort type is necessary as foundational data regarding post‐HDP biology is drastically lacking. We were not subject to batch effects as all samples were run simultaneously. We employed robust statistical methods, including corrected *p* values for multiple comparisons, and orthogonal accounting for underlying cardiovascular risk by adjusting for age, BMI, diabetes, and chronic hypertension, and including a sensitivity analysis excluding individuals with hypertension or diabetes. Although we found significant differences in several key CVD‐associated proteins, additional studies in larger cohorts are needed to further advance our understanding of post‐HDP cardiovascular biology.

### 4.1 Perspectives

The prevalence of HDP is on the rise. The prevalence of HDP among US delivery hospitalizations in 2019 was close to 16%, up from 13% just two years prior [70]. At the other end of the life course, CVD remains the leading worldwide cause of mortality in women [1]. With the well‐established connection between HDP and CVD and the combined global burdens of these two entities, stratification of cardiovascular risk in the intervening years, after HDP but before evident CVD, is urgently needed. We present the first cohort demonstrating significant elevations, by orders of magnitude, in several key proteins known to be implicated in CVD in the few years following an HDP pregnancy compared to matched participants, and after controlling for critical confounders. This provides molecular evidence of post‐HDP cardiovascular risk and that exposure to the HDP pregnancy itself may have measurable impact. These circulating biomarkers may represent promising targets for identifying those at the highest risk for developing CVD and informing interventions that mitigate CVD event risk in this high‐risk population.

## CONFLICT OF INTEREST STATEMENT

The authors declare no conflicts of interest.

## Supporting information



Supporting Information

## Data Availability

De‐identified clinical meta data and raw values for protein concentrations have been made publicly available at Dryad and can be accessed at https://doi.org/10.5061/dryad.rn8pk0pmc.
