# Supplementary material for: Higher Levels of Cardiovascular Biomarkers Following Hypertensive Compared to Normotensive Pregnancy
Source: Pregnancy (Hoboken). Author manuscript; Available in PMC 2026 Apr 15. (PMC13078767; doi:10.1002/pmf2.70064)
Supplement: Supplementary Material [file NIHMS2164019-supplement-Supplementary_Material.docx]

**SUPPLEMENTARY MATERIALS**

**SUPPLEMENTARY TABLE 1**

|  |  | **Univariate Analysis** | **Multivariable Analysis** | | |
| --- | --- | --- | --- | --- | --- |
| **Protein** | **Variable** | **Estimate** | **Estimate** | **Std. Error** | ***P* value** |
| Fibrinogen | Post-HDP | 0.379 | 0.417 | 0.065 | 3.47E-08 |
|  | Maternal age | -0.002 | 0.003 | 0.006 | 0.640 |
|  | BMI | -0.007 | -0.014 | 0.005 | 0.013 |
|  | DM | 0.036 | 0.146 | 0.093 | 0.120 |
|  | cHTN | 0.025 | 0.089 | 0.097 | 0.361 |
|  |  |  |  |  |  |
| Fetuin-A36 | Post-HDP | 0.198 | 0.202 | 0.032 | 4.89E-08 |
|  | Maternal age | -0.004 | -0.002 | 0.003 | 0.522 |
|  | BMI | -0.001 | -0.004 | 0.003 | 0.143 |
|  | DM | 0.057 | 0.054 | 0.045 | 0.240 |
|  | cHTN | 0.017 | 0.027 | 0.048 | 0.569 |
|  |  |  |  |  |  |
| L-selectin | Post-HDP | 0.324 | 0.321 | 0.069 | 2.32E-05 |
|  | Maternal age | -0.014 | -0.010 | 0.006 | 0.116 |
|  | BMI | -0.012 | -0.013 | 0.006 | 0.025 |
|  | DM | -0.028 | 0.096 | 0.099 | 0.339 |
|  | cHTN | -0.182 | -0.106 | 0.103 | 0.308 |
|  |  |  |  |  |  |
| AGP | Post-HDP | 0.349 | 0.375 | 0.083 | 3.60E-05 |
|  | Maternal age | -0.001 | 0.004 | 0.008 | 0.579 |
|  | BMI | 0.004 | -0.008 | 0.007 | 0.247 |
|  | DM | 0.272 | 0.342 | 0.119 | 0.006 |
|  | cHTN | 0.097 | 0.060 | 0.124 | 0.631 |

**Supplementary Table 1. Univariate and multivariable linear regression coefficients.**

Coefficients for each variable presented as the -log_10_(estimate) from linear regression analysis with each variable input initially as a univariate analysis (unadjusted) or in the multivariable analysis (estimate). For the multivariable analysis, the standard error and the P value are included for each distinct variable analyzed. Variables include diabetes (DM), presence or absence of chronic hypertension (cHTN), earliest BMI in the most recent pregnancy (BMI), and maternal age at the time of sample collection.

**SUPPLEMENTARY FIGURES**

**Supplementary Figure 1**

**
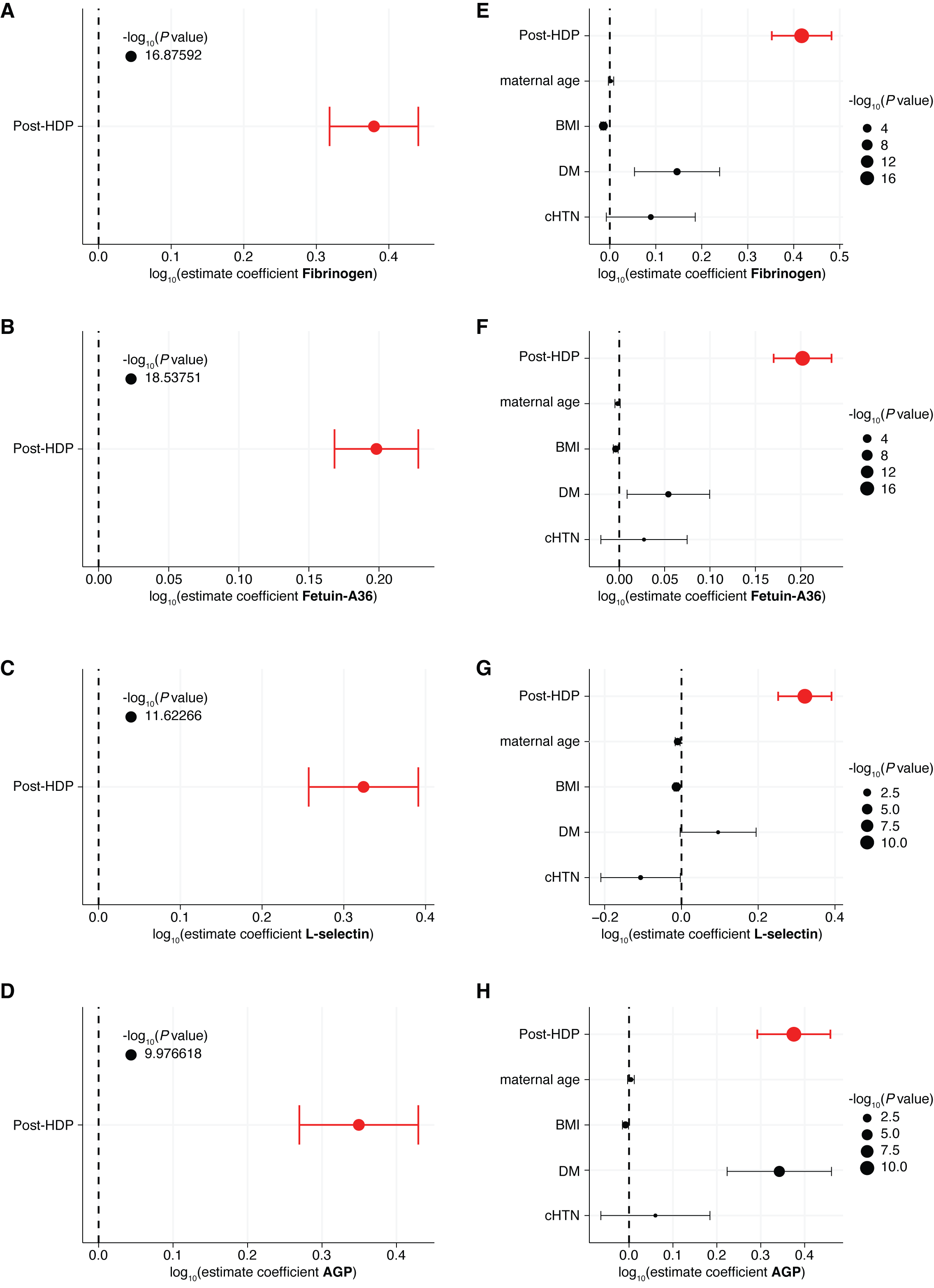
**

**Supplementary Figure 1. Association of HDP history with CVD-associated proteins in both univariate and multivariable regression analysis controlling for key confounders.**

(**A-D**) Univariate linear regression model assessing contribution of HDP status (i.e., post-Normal vs. post-HDP) on plasma protein levels of fibrinogen (**A**), fetuin-A36 (**B**), L-selectin (**C**), or AGP (**D**).

(**E-H**) Multivariable regression model assessing potential confounders to plasma protein levels of fibrinogen (**E**), fetuin-A36 (**F**), L-selectin (**G**), or AGP (**H**) by HDP status (i.e., post-Normal vs. post-HDP). Confounders included earliest BMI of the most recent pregnancy, maternal age at sample collection, diabetes, and chronic hypertension. Each point scaled to -log_10_(*P* value) with error bars representing the standard error of the estimated coefficient.

**Supplementary Figure 2**


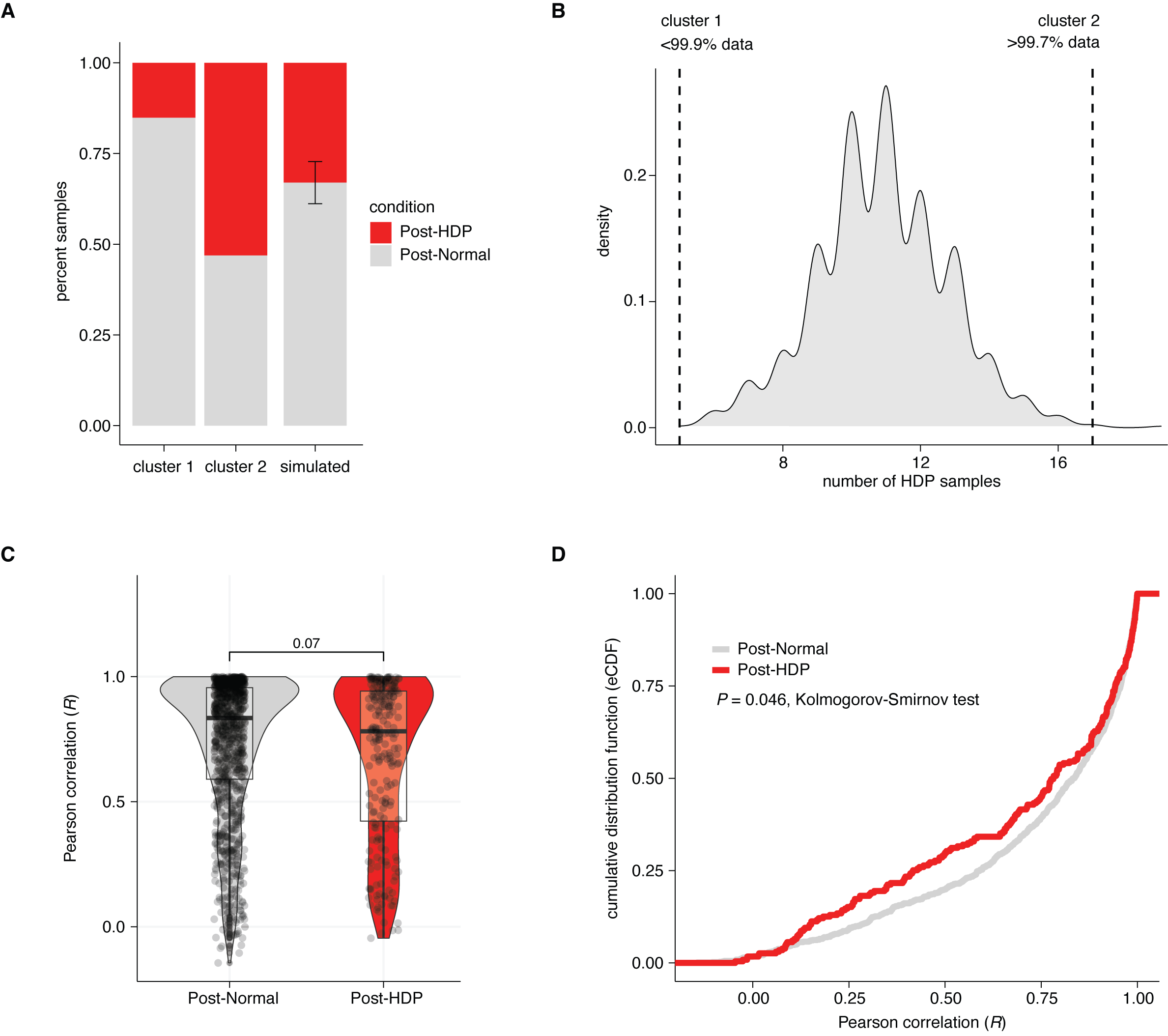


**Supplementary Figure 2. Quantifying global patterns of CVD associated protein expression following HDP.**

(**A**) Stacked bar plots of the fraction of Post-HDP (red) and Post-Normal (gray) samples present in cluster 1 and cluster 2 (first branch from X-axis dendrogram Figure 3A) and simulated data from 1000 random samples of 33 samples from our cohort. Error bars reflect the standard deviation.

(**B**) Density plot of all 1000 random samples reflecting the number of HDP samples of 33 randomly selected. Vertical dashed lines indicate the actual observed data for cluster 1 and cluster 2.

(**C**) Schematic demonstrating example scatter plot comparing all CVD protein expressions in two distinct patients. For each comparison, the Pearson correlation coefficient (R) is extracted, and then the process is repeated comparing each participant’s global CVD expression profile to each other participant within its respective group (Post-Normal vs. Post-HDP).

(**D**) Estimated cumulative distribution function (eCDF) of the intra-group Pearson correlation coefficients for Post-HDP (red) or Post-Normal (gray). *P* value from a two-sided Kolmogorov-Smirnov test.

**Supplementary Figure 3**


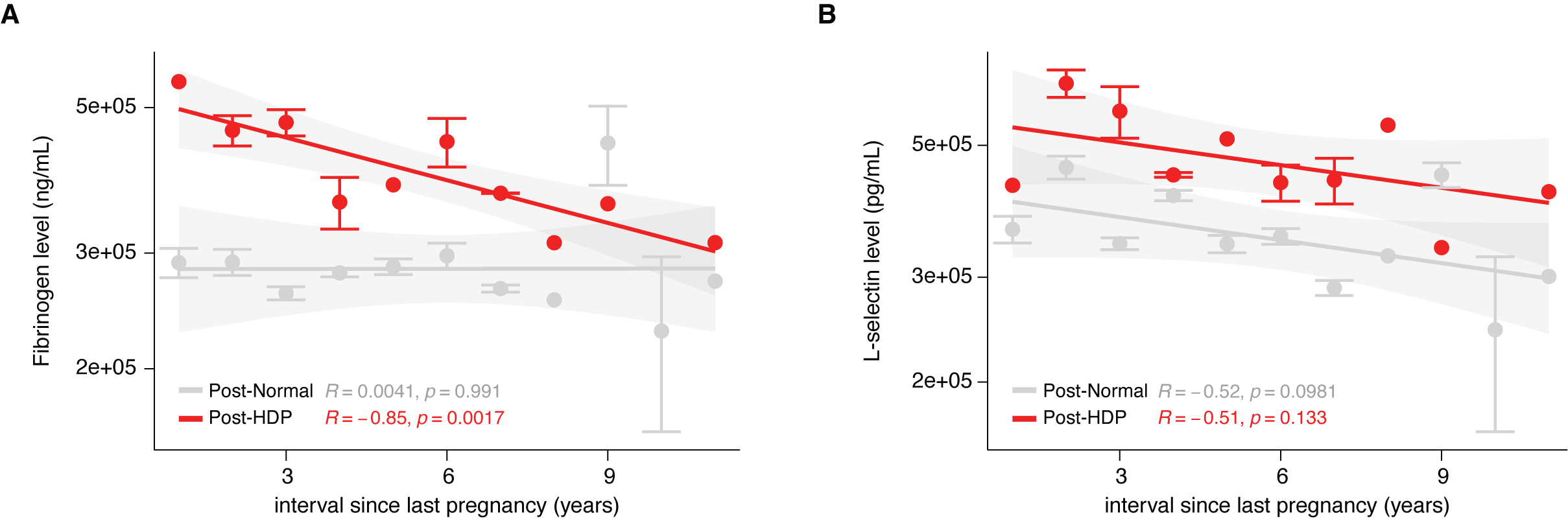


**Supplementary Figure 3.** **Time since most recent pregnancy and plasma protein levels**

(**A**) Mean protein values of fibrinogen levels per condition (Post-Normal or Post-HDP, grouped by years since last pregnancy) compared to time since last pregnancy. Error bars reflect standard error of the mean and grey bands represent the 95% confidence interval of the linear regression. *R* and *P* value from Pearson correlation.

(**B**) Mean protein values of L-selectin levels per condition (Post-Normal or Post-HDP, grouped by years since last pregnancy) compared to time since last pregnancy. Error bars reflect standard error of the mean and grey bands represent 95% confidence interval of the linear regression. *R* and *P* value from Pearson correlation.

*Note that samples are from individual participants and are not measures within the same participant.*

**Supplementary Figure 4**


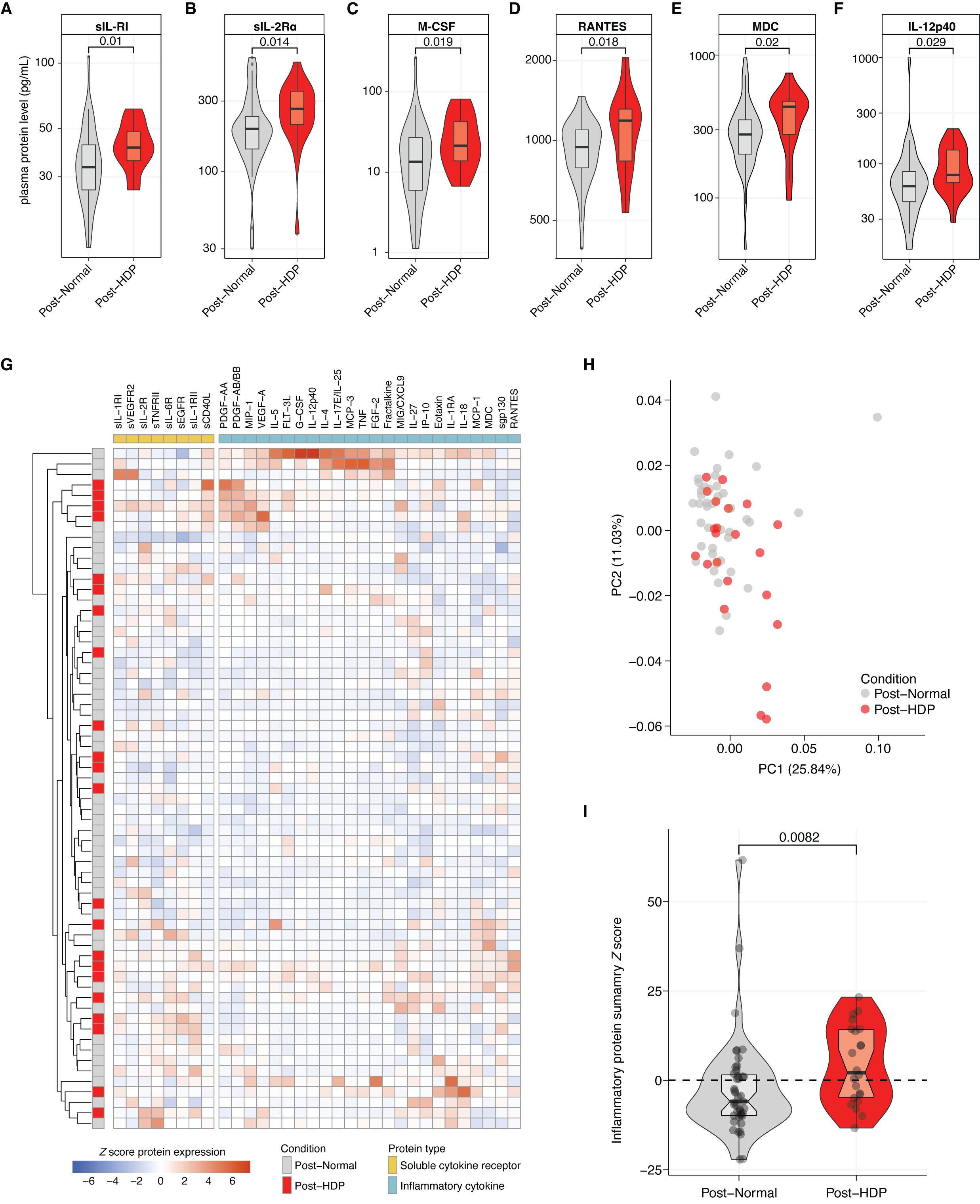


**Supplementary Figure 4. Differential expression of the inflammatory markers in our cohort.**

(**A-F**) Violin plot of sIL-RI, sIL-2R, M-CSF, RANTES, MDC, and IL-12p40 protein levels (ng/mL) in plasma from individuals following a normotensive pregnancy (Post-Normal) or pregnancy meeting HDP criteria (Post-HDP). *P* value from a two-sided Wilcoxon Rank Sum Test.

(**G**) Heatmap of *Z* scores of soluble cytokine receptors and inflammatory cytokines expression with columns reflecting an individual protein and rows corresponding to an individual plasma sample. Samples colored by condition as Post-Normal (gray) or post-HDP (red). Proteins colored by protein panel as either a soluble cytokine receptor (yellow) or an inflammatory cytokine (blue). For all proteins analyzed in our assays only proteins with detectable protein values in every sample in included in the heatmap (N = 32 proteins).

(**H**) PCA plot of Post-Normal (gray) and Post-HDP (red) protein values for plasma protein expression values of soluble cytokine receptors and inflammatory cytokines.

(**G**) Violin plot of soluble cytokine receptors and inflammatory cytokines summary *Z* score calculated by summing *Z* scores of all associated protein levels reflected in panel **G** in plasma from individuals following a normotensive pregnancy (Post-Normal) or pregnancy meeting HDP criteria (Post-HDP). *P* value from a two-sided Wilcoxon Rank Sum Test.
